# Platinized Graphene/ceramics Nano-sandwiched Architectures and Electrodes with Outstanding Performance for PEM Fuel Cells

**DOI:** 10.1038/srep16246

**Published:** 2015-11-05

**Authors:** Xu Chen, Daping He, Hui Wu, Xiaofeng Zhao, Jian Zhang, Kun Cheng, Peng Wu, Shichun Mu

**Affiliations:** 1 State Key Laboratory of Advanced Technology for Materials Synthesis and Processing, Wuhan University of Technology, Wuhan 430070, China

## Abstract

For the first time a novel oxygen reduction catalyst with a 3D platinized graphene/nano-ceramic sandwiched architecture is successfully prepared by an unusual method. Herein the specific gravity of graphene nanosheets (GNS) is tailored by platinizing graphene in advance to shorten the difference in the specific gravity between carbon and SiC materials, and then nano-SiC is well intercalated into GNS interlayers. This nano-architecture with highly dispersed Pt nanoparticles exhibits a very high oxygen reduction reaction (ORR) activity and polymer electrolyte membrane (PEM) fuel cell performance. The mass activity of half cells is 1.6 times of that of the GNS supported Pt, and 2.4 times that of the commercial Pt/C catalyst, respectively. Moreover, after an accelerated stress test our catalyst shows a predominantly electrochemical stability compared with benchmarks. Further fuel cell tests show a maximum power density as high as 747 mW/cm^2^ at low Pt loading, which is more than 2 times higher than that of fuel cells with the pristine graphene electrode.

Polymer electrolyte membrane (PEM) fuel cells are a promising electrochemical device via a direct conversion of hydrogen energy into electricity^1^. However, the very low stability of Pt-based catalysts and utilization of Pt in fuel cells have seriously blocked their commercialization. At present, carbon black (e.g., Vulcan XC-72R), as predominant supports for Pt-based catalysts, is extensively used in PEM fuel cells^2^. Unfortunately, sensitive electrochemical oxidation is suffered under harsh work conditions at cathodes for fuel cells^3^. This leads to irreversible aggregation of noble metal (e.g., Pt) nanoparticles (NPs) and subsequent serious degradation of electrochemical surface area (ECSA)^4^. Consequently, seeking the advanced support materials to promote the catalytic activity and durability of electrocatalysts has become a research focus^5^. Recently, graphene nanosheets (GNS) have attracted attention as a unique 2D material with really large theoretical specific surface area (2630 m^2^g^−1^), high electrical conductivity and excellent chemical stability^6^. Thus, an intensive effort has focused on GNS as highly efficient catalyst supports, and some exciting results have been reported^5^,^7^,^8^. However, due to the nature of 2D materials, GNS readily tend to restack through the strong π−π interaction^7^, which hinders a substantial amount of catalytic sites on catalytic nanocrystals and sets a higher resistance for the diffusion of reactant species, leading to the retarded catalytic reaction and unusually low Pt utilization^9^.

Some attempts have been made to prevent the re-stacking of GNS, including the combination of GNS with other carbon building blocks, such as carbon nanotubes, carbon nanospheres, fullerene and carbon nanofibers^10^. For example, Zhang *et al.* have reported a 3D CNT/graphene structure with CNT pillars grown in between graphene layers^11^. Our previous work has presented a quasi-sandwiched graphene/carbon nanosphere architecture with enhanced electrochemically accessible surface area and mass transfer^9^. However, such carbon building blocks can be electrochemically eroded in the sensitively oxidized environment of PEM fuel cells. Hence, conductive nano-ceramics (such as WC and TiB_2_) have raised considerable interests as alternative support materials for metal catalysts due to their outstanding oxidation and acid corrosion resistance^12^,^13^,^14^. We have reported nano-boron carbide (B_4_C), nano-silicon carbide (SiC), titaniumdiboride (TiB_2_) can be taken as example to stabilize catalyst supports for PEM fuel cells^15^,^16^,^17^.

Nevertheless, differently from the previous architectures, the presence of a big difference in the specific gravity between nano-ceramics and graphene (~1.06 g/cm^3^)^18^ or graphene oxide (~2.2 g/cm^3^)^19^ badly prevents the nano-ceramic from being homogeneously embedded into gaps in between carbon nanosheets within liquid solutions and subsequently co-deposition^20^. For this purpose, here we for the first time report a novel approach to assembling such GNS/ceramics structures by platinizing GNS in advance to increase the specific gravity of carbon materials, as shown in Fig. 1. As anticipated, the 3D structural hybrid, as a highly efficient support of Pt NPs, reveals a remarkably improved electrocatalytic property and PEM fuel cell performance by increasing surface area and mass transfer in the novel graphene electrode.

## Results

### Material and structural properties

As shown in Fig. 2a,b, after introducing nano-SiC into GNS interlayers, a novel 3D sandwiched architecture is achieved, where GNS are adequately separated by nano-SiC layers. As a result, the re-stacking of GNS is effectively impeded. Nano-SiC between graphene interlayers can act as nanoscale pillars to prevent the aggregation of GNS and to increase the spacing between adjacent carbon nanosheets, avoiding face-to-face van der Waals contact of neighboring nanosheets. In this unique architecture, double side surfaces of GNS can be accessed, and the enlarged interlayer spacing can provide a high density of between-plane electrolyte diffusion channels that speed up the transport of reaction species. On contrast, for Pt/RGO, it exhibits a typically layered structure with crumpled surfaces (Fig. S1 a and b) because the 2D RGO nanosheet is radically inclined to re-stack in light of the strong π−π interaction, greatly reducing the specific surface area of graphene.

As shown in Fig. S1c and b, the average particle size of Pt NPs, with a uniform dispersion on RGO surfaces, is 2.62 nm, which can be attributed to the anchoring function of oxygen-containing functional groups on RGO. Fig. 2c,d present inserting nano-SiC NPs into GNS does not affect the homogeneous dispersion of Pt NPs on RGO in Pt-RGO/SiC. The lattice spacing of 0.22 and 0.25 nm corresponds to Pt (1 1 1) and SiC (1 1 1) facets. The average particle size of nano-SiC is 50 nm and Pt NPs have a very narrow particle size with diameters in the range of 1 to 3 nm (~1.98 nm in average). Moreover, the size of Pt NPs in the commercial Pt/C catalyst is 2.96 nm (Fig. S2). Note that, some partially covered SiC NPs by the Pt dispersed graphene can be found in TEM observation (Fig. S3), where no Pt NPs are present on the uncovered part of SiC NPs. This evidences no dispersed Pt NPs directly distribute on SiC surfaces.

Fig. 3a exhibits XRD patterns of Pt/RGO and Pt-RGO/SiC. Broad hump lines with maxima at a 2θ value of 23–25° can be observed for Pt/RGO, which are attributed to the (002) diffraction signal from the graphite crystal^21^. It can be found that the XRD diffraction peak of the carbon (002) facet almost disappears for Pt-RGO/SiC, indicating the well-done exfoliation of carbon nanosheets caused by the intercalation of nano-SiC into graphene layers^9^. Instead, according to our previous work^20^, due to presence of the big difference in the specific weight, the nano-ceramic is normally wedged into GNS stacks while not into GNS interlayers, and then no shift of the XRD diffraction peak of carbon (002) facet peaks can be observed after wedging of ceramics, indicating the spacing of GNS interlayers is almost intact. This result is in good agreement with our assumption that platinizing GNS could promote the intercalation of nano-ceramics into GNS in liquid solutions by means of the decreased difference in the specific gravity between GNS and ceramics.

Fig. 3b displays Raman spectra of both Pt/RGO and Pt-RGO/SiC. The peaks at 1585 and 1348 cm^−1^ correspond to the G band related to the vibration of sp^2^-bonded carbon atoms and the D band associated with the vibrations of carbon atoms having dangling bonds in plane terminations of disordered graphite^22^. The increased intensity ratios of D band to G band (ID/IG) from Pt/RGO (0.92) to Pt-RGO/SiC (1.06) show a more disordered structure due to the exfoliation of nanosheets after inserting nano-SiC into GNS^23^. This result is in agreement with the earlier observation and analysis from SEM/TEM images and XRD patterns.

As shown in Fig. S4, the BET surface area of the Pt/RGO is about 157.9 m^2^ g^−1^, by contrast, Pt-RGO/SiC reaches 395.5 m^2^ g^−1^ which is more than two times of that of Pt/RGO. This indicates the successful insertion of nano-SiC NPs into graphene interlayers, which is in accordance with the analytical results from SEM/TEM images, XRD patterns and Raman spectra.

As shown in Fig. S5, EIS plots of all samples contain partially overlapped semicircles where the charge transfer resistance (R_ct_) and the uncompensated solution, contact and bulk resistance (R_el_) in different supports are present. The R_el_ value is very close to each other for different electrodes (0.5 ~ 0.7 Ω) although it shows the sequence: Pt-RGO/SiC > Pt/RGO > Pt/C. Moreover, the values of R_ct_ for different electrodes is Pt-RGO/SiC (8.02 Ω) > Pt/RGO (7.51 Ω) > Pt/C (7.12 Ω). The results exhibit the electric conductivity of the Pt-RGO/SiC catalyst is very close to Pt/RGO and Pt/C. The excellent conductivity of Pt-RGO/SiC can facilitate the improvement of activity and stability towards oxygen reduction reaction (ORR).

### Electrocatalytic Activities

Fig. 4a exhibits CV curves of catalysts recorded at room temperatures from 0 to +1.2 V vs. RHE at a scan rate of 50 mVs^−1^. As seen in Fig. 4b, the electrochemical surface area (ECSA) was calculated from measuring the hydrogen adsorption-desorption region assuming a value of 210 uC cm^−2^ for the adsorption of a hydrogen monolayer on Pt.^24^ Significantly, our Pt-RGO/SiC catalyst reveals an unusually high ECSA (112 m^2^ g^−1^) which increases by 40 and 87% in comparison with Pt/RGO (80 m^2^ g^−1^) and Pt/C (60 m^2^ g^−1^), respectively. Furthermore, the potential window between +0.3 and +0.8 V vs. RHE is an indicator of capacitive current decided by the electrochemically accessible area where the electrolyte can reach the internal micropores of carbon materials^25^. Our catalyst reveals a significantly broader electrochemical specific double layer than that of Pt/RGO (Fig. 4a), indicating the increased specific surface area and the greater accessibility to the electrolyte and charged ions^26^. This result demonstrates a dramatic improvement in electrochemically accessible surface area for Pt-RGO/SiC, due to its unique 3D nano-sandwiched structure.

The kinetic current can be calculated from ORR polarization curves according to the Koutecky-Levich equation (Fig. 4c)^27^. As shown in Fig. 4d, the mass activity of Pt-RGO/SiC (174 A g^−1^) is 1.6 and 2.4 times versus Pt/RGO (112 A g^−1^) and Pt/C (72 A g^−1^), respectively, indicating a greatly improved ORR activity for Pt-RGO/SiC. In addition, the Pt-RGO/SiC electrode exhibits a higher limiting current density (5.9 mA cm^−2^) than Pt/RGO (5.15 mA cm^−2^) and Pt/C (5.5 mA cm^−2^). This proves that the sandwiched structure benefits mass transfer, leading to low concentration polarization. This finding is also in good accordance with the single cell test results (see later).

### Electrocatalytic Stabilities

The ECSA loss of Pt catalysts over different electrochemical oxidation cycles is shown in Fig. 5a, and increases with the number of cycles under the same electrochemical acceleration test (Fig. S6). After 10000 cycles, only 20.7% of initial ECSA of Pt/C remains, while 28.2% is for Pt/RGO. But surprisingly, Pt-RGO/SiC retains 40.3% of initial ECSA after 10000 cycles. Such findings clearly demonstrate that our new catalyst has an electrochemical stability up to 1.9 and 1.4 times higher than that of Pt/C and Pt/RGO, respectively, under the same AST conditions.

To reveal the mechanism of the stability, the structure change of catalysts after AST was investigated by TEM observation (Fig. 5b–e). The particle size distributions of Pt NPs for all catalysts were measured for more than 100 particles in each sample. After 10000 CV cycles the size of Pt NPs of all samples increases, indicating the occurrence of agglomeration for metal NPs. A further comparison strongly suggests the particle size of Pt NPs in Pt-RGO/SiC is much smaller than those in Pt/RGO and Pt/C samples after AST. In the case of Pt/RGO, a massive decrease of Pt NPs can be found in Fig. 5b, where the average particle size increases from 3.0 to 7.5 nm (Fig. 5c). In contrast, relatively low agglomeration of Pt NPs occurs for Pt-RGO/SiC (Fig. 5d) with a more sluggish increase in the particle size from 2.0 to 6.5 nm (Fig. 5e). As a reference, serious agglomeration of Pt NPs (from 3.0 to 8.0 nm) appears for Pt/C (Fig. S7).

### PEM Fuel Cell Performance

As shown in Fig. 6a,b, the catalyst layer in Pt-RGO/SiC electrodes for PEM fuel cells shows a porous property (Fig. 6a), and the sandwiched architecture in catalysts is also preserved (Fig. 6b) even undergoing a radical electrode preparation process including the high-speed agitation of ink, the hot-pressing treatment of CCM and subsequently screwing the single cell. By contrast, for the Pt/RGO electrode a re-stacking of graphene and a relative compact catalyst layer structure forms (Fig. 6c,d).

Fig. 6e,f exhibit the fuel cell performance using graphene electrodes by single cell tests. Under the same Pt loading, the fuel cell voltage output presented in polarization curves for Pt-RGO/SiC electrodes is notably higher than that for Pt/RGO electrodes. Especially, at the concentration polarization region (where the cell works at lager current densities even up to limiting current), the former possess voltage output of 197 mV than the latter, indicating an excellent mass transfer property for the Pt-RGO/SiC electrode owing to the porous catalyst layer (Fig. 6a,b) and the unique catalyst with the nano-sandwiched architecture (Fig. 2) in electrodes. While the low voltage output performance for Pt/RGO electrodes can be attributed to the restacking GNS structure that heavily blocks the mass transfer.

Significantly, it is very interesting that the Pt-RGO/SiC electrode maintains much higher performance than that of the Pt/RGO electrode even under lower humidity conditions decreased from 100 to 50% RH. The maximum power density for Pt-RGO/SiC electrodes reaches 747 and 603 mW cm^−2^ corresponding to 100 and 50% humidification, while for the Pt/RGO electrode it only attains 343 and 301 mW cm^−2^, respectively. Those demonstrate the enlarged spacing of such nano-sandwiched architectures could reserve much more water in comparison with the Pt/RGO electrode. The increased water uptake leads to the high proton conductivity for ionomers in both catalyst layers and membranes under low humidification, greatly improving fuel cell performance under low humidity or relative dry conditions.

## Discussion

From the results of SEM, TEM XRD, Raman and BET measurements, it can be seen that the nano-ceramic particles have been homogenously intercalated into grapehene by platinizing GNS in advance due to the decreased difference in the specific gravity between GNS and ceramics. Instead, a serious agglomeration of nano-ceramics with a wedge structure can be observed (Fig. S8). Such typical GNS/ceramics/GNS sandwiched architecture is effective to avoid restacking of GNS caused by the strong π−π interaction and very favorable for enhancing the electrochemical properties of GNS based catalysts: 1) the enlarged spacing between nanosheets due to the intercalation of nano-SiC particles with a good conductive property, leads to the improved diffusion of electrolyte and transport of the reaction species; 2) Pt NPS distributed on both nanosheets can be fully exposed, this is because such structure can keep nanosheets stable and avoid folding of nanosheets as well as obscuring of Pt NPs, enhancing the usage of Pt NPs. As results shown, our Pt-RGO/SiC catalyst presents an abnormally high ECSA of 112 m^2^ g^−1^ which increases by 40 and 87%, respectively, in comparison with the Pt/RGO and commercial Pt/C catalysts. Significantly the mass activity of Pt-RGO/SiC (174 A g^−1^) is improved greatly, which is 1.6 and 2.4 times versus Pt/RGO and Pt/C, respectively. The high mass transfer capability and activity of our catalyst contribute to the excellent PEM fuel cell performance. Especially, the enlarged spacing between GNS in comparison with that of the pristine GNS increases the water retention in catalyst layers, which is useful to improve the fuel cell performance under low humidification conditions.

The GNS/ceramics sandwiched architecture where GNS are pillared by durable nano-SiC NPs endows the 3D composite with a higher structural stability, preventing the nanosheet from rapid re-stacking or folding during electrochemical accelerated stress test (AST). In addition, the presence of nano-SiC NPs in between GNS improves the Pt dispersion because they can block the migration and aggregation of Pt NPs during AST^28^. These lead to the sandwiched structure catalyst retains outstanding stability of ECSA even after 10000 CV cycles to compare with Pt/C and Pt/RGO. This is critical for promoting the long-term performance of PEM fuel cells. In contrast, for Pt/RGO, the readily folded pristine nanosheets can cover the active sites of Pt and separate electrolyte from the reaction system, accelerating inactivation of Pt when used as catalyst supports.

In summary, a nano-sandwich structured platinized graphene/nano-SiC hybrid was successfully prepared by a simple and unique method. Instead of the strong re-stacking trend for conventional graphene nanosheets, with the intercalation of nano-SiC into reduced graphene oxide (RGO), the obtained 3D Pt-RGO/SiC architecture is endowed with a large surface area and high mass transfer for reaction species. As a result, for half cells the new catalyst shows a higher activity in electrochemical surface area and oxygen reduction activity, as well as a much better stability compared to the Pt/RGO and Pt/C catalysts. Significantly, the single fuel cell tests exhibited the maximum power density of 747 mW cm^−2^ for the graphene electrode of Pt-RGO/SiC at low Pt loading, which was two times more than that of the pure graphene electrode of Pt/RGO. Therefore our new catalyst holds a tremendous promise for applications in PEM fuel cells and other fields.

## Methods

**Figure 1:**
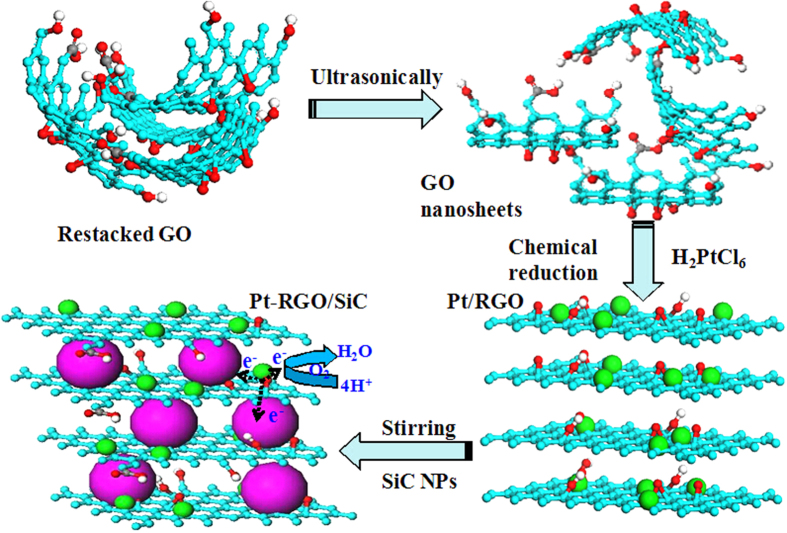
Illustration of the formation of a layer-by-layer Pt-RGO/SiC architecture including the steps of restacked GO, dispersed GO nanosheets in a solution, deposition of Pt nanoparticles on RGO and insertion of nano-ceramics into dispersed Pt/RGO layers.

**Figure 2:**
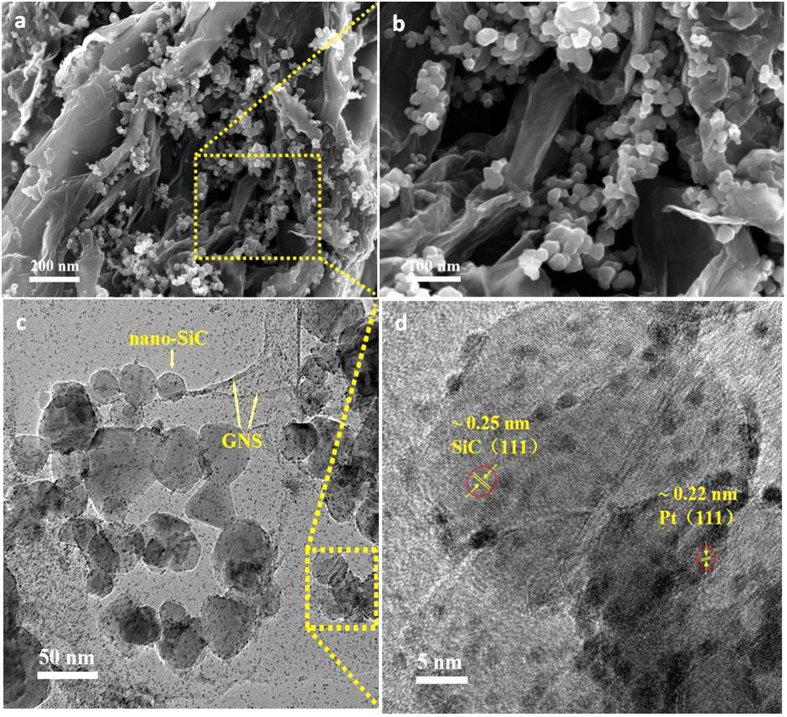
SEM (**a,b**) and TEM (**c,d**) images of the Pt-RGO/SiC catalyst.

**Figure 3:**
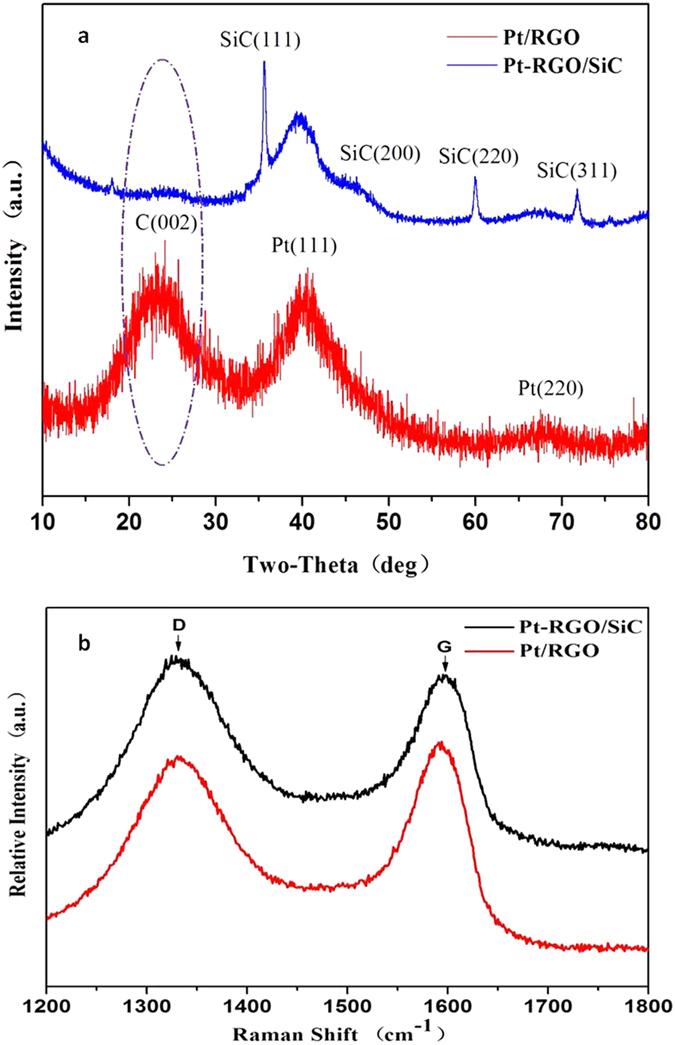
XRD spectra (**a**) and Raman spectra (**b**) of Pt/RGO and Pt-RGO/SiC catalysts.

**Figure 4:**
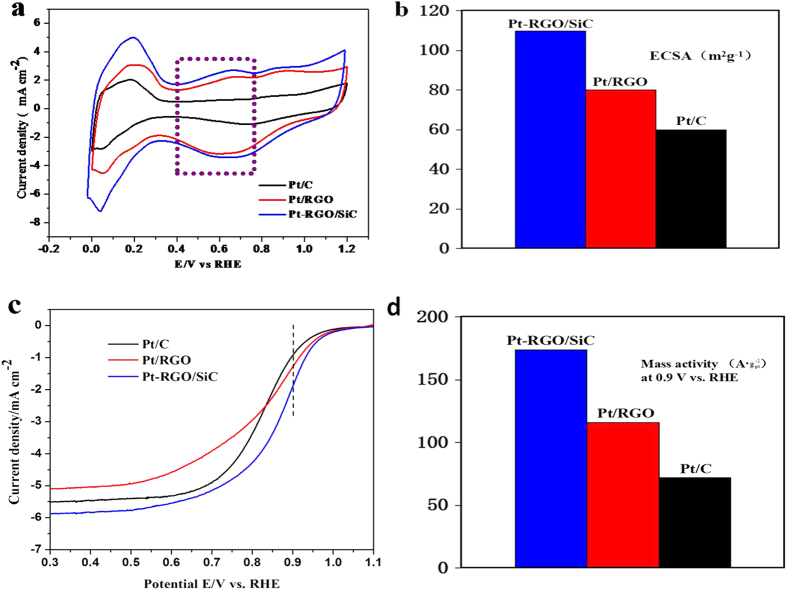
CV curves (**a**) and ECSA (**b**) of Pt-RGO/SiC, Pt/RGO and commercial Pt/C catalysts; current-potential polarization curves for ORR (**c**) and mass activities at +0.9 V vs. RHE (**d**) in 0.1 M HClO_4_ solution.

**Figure 5:**
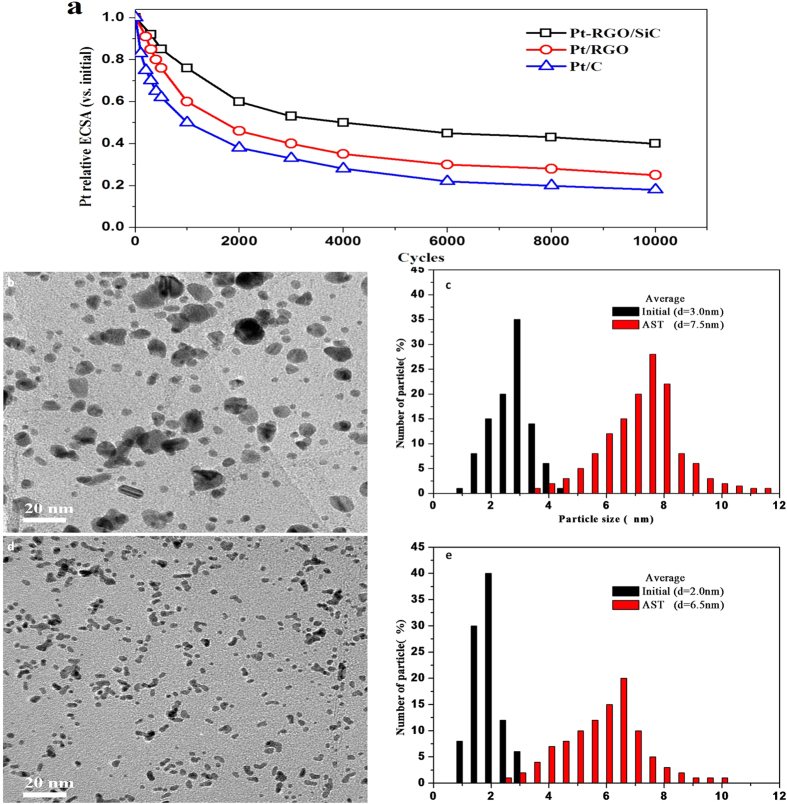
Changes of ECSA (**a**) as a function of the number of potential cycles; TEM images of the Pt/RGO (**b**) and Pt-RGO/SiC (**d**) catalysts after AST, and Pt particle size distributions of Pt/RGO (**c**) and Pt-RGO/SiC (**e**) before (black) and after (red) AST.

**Figure 6:**
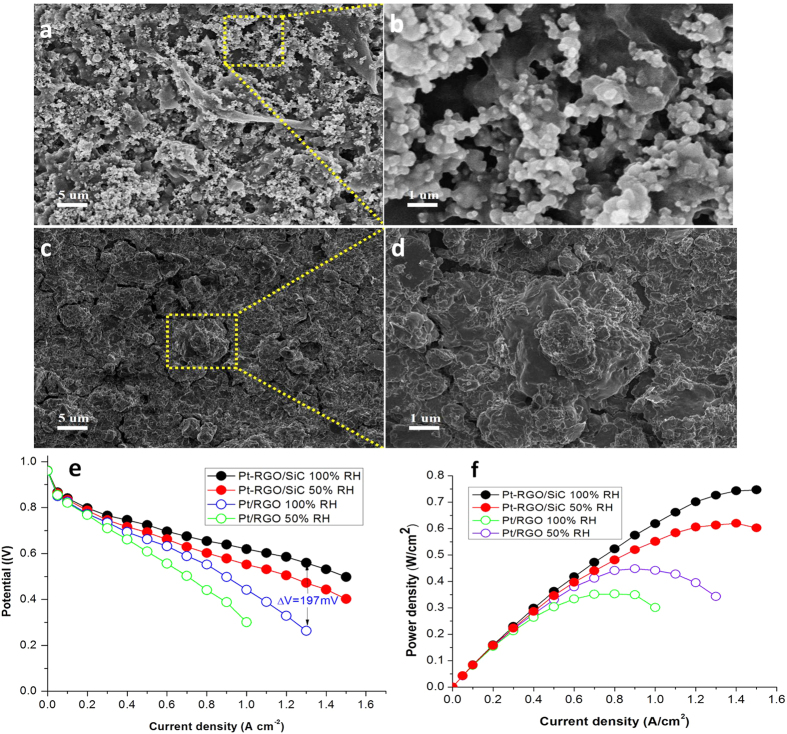
A SEM image of catalyst layers at cathode electrodes applied in PEM fuel cells for Pt-RGO/SiC (**a**) and a selective-area magnified image (**b**); A SEM image of catalyst layers at cathode electrodes for PEM fuel cells for Pt/RGO (**c**) and a selective-area magnified image (**d**); Fuel cell performance by single cell tests Polarization curves (**e**) and power density (**f**) for Pt-RGO/SiC and Pt/RGO electrodes.

Graphene oxide (GO) was synthesized from natural graphite flakes through a modified Hummer’s method described previously^29^. The process of synthesizing sandwiched Pt-RGO/SiC hybrids is depicted in Fig. 1 where Pt NPs were firstly homogeneously deposited on the reduced GO (RGO) by ethylene glycol (EG) reduction^30^. Sixty milligram of GO nanocomposites was added to EG solution and followed by an ultrasonic treatment for 30 min, and then transferred into a round bottom flask. Afterwards, the Pt precursor H_2_PtCl_6_·6H_2_O (Sinopharm Chemical Reagent Co., Ltd.) solution was added dropwise into the GO suspension under vigorous stirring. pH of the solution was adjusted to 10–12 using 1.0 M of NaOH aqueous solution, and then the mixture was heated under reflux at 150 °C for 3–4 h to ensure Pt NPs were completely produced and GO was reduced to RGO. After that, twenty milligram of nano-SiC with an average particle size of 50 nm was mixed with Pt/RGO aqueous suspension, after stirring overnight, the mixture was filtered and washed with deionized water. The obtained catalyst was dried in a vacuum oven at 80 °C for 8 h. For comparison purposes, RGO supported Pt catalysts (Pt/RGO) were synthesized following a similar procedure and the commercial Hispec 3000 catalyst (20 wt.% Pt supported on carbon black) purchased from Johnson Matthey was employed.

Microstructures of the as-prepared hybrid were analyzed using JEOL 2010 high-resolution transmission electron microscope (HRTEM), JEOL JEM 6700 scanning electron microscope (SEM) operated at 10 kV, as well as X-ray diffraction with Cu K_α_ source (λ = 1.54056 Å) at a scan rate of 5° min^−1^ from 10 to 80°. Raman spectroscopy was carried out on a Renishaw using Ar ion laser with an excitation wavelength of 514.5 nm.

Electrochemical experiments were carried out using the methods described by Gasteiger^31^, Takahashi^32^, Curnick^33^ and Garsany^34^ performed at (298 ± 1) K in a 3electrode cell set up in a Faraday cage, using an Autolab PGSTAT30 potentiostat (EcoChemie B.V, Holland) and a RDE setup (Pine Instruments). The background electrolyte was 0.1 M double distilled ultrapure perchloric acid (HClO4) prepared from 70% (Sinopharm Chemical Reagent Co., Ltd). A saturated calomel electrode and a platinum wire were used as a reference electrode and a counter electrode, respectively. Herein, all the potentials were present on the scale of the reversible hydrogen electrode (RHE). All glassware was subjected to a rigorous cleaning procedure involving soaking in sulfuric acid for eight hours, followed by thoroughly rinsing and boiling in ultrapure water to ensure minimal residual impurities. Moreover, electrochemical impedance spectroscopy (EIS) was measured by an EC-lab SP300 frequency response analyzer and the frequency ranged from 1 MHz to 1 Hz.

The dispersed catalysts were deposited on glassy carbon according to Pollet *et al.*^35^,^36^. Three milligram catalyst (20 wt.% Pt/C, 20 wt.% Pt/RGO and 20 wt.% Pt-RGO/SiC) powders were mixed with 500 uL isopropanol, 480 uL ultrapure water (R = 18.2 MΩ) and 20 uL 5 wt.% Nafion (DuPont Co., Ltd.) solution in an ultrasonic bath (Grant Instruments, 38 kHz) for 0.5 h. And then the working electrode was prepared by dropping of 5 μL catalytic ink on a freshly polished glassy carbon disk (d = 5.0 mm), leading to a total Pt loading of approximately 15 μgPtcm^−2^. The solvent was then evaporated in a stream of nitrogen at room temperature.

Before ECSA experiments, the solutions were deoxygenated by bubbling nitrogen for 20 min and then the electrode was conditioned by potential cycling for 50 cycles between 0 V and +1.2 V vs. RHE at 100 mV s 1. Cyclic voltammograms (CVs) were carried out in an N_2_-purged. 1 M HClO_4_ electrolyte solution at a scan rate of 50 mV s^−1^ in a potential range of 0 to +1.2 V vs. RHE at room temperature. Then, the half-cell oxygen reduction reaction (ORR) of catalysts was evaluated by a rotating disk electrode technique in 0.1 M HClO_4_ electrolyte at a sweep rate of 10 mV s 1and a speed of 1600 rpm at room temperature. An electrochemical accelerated stress test (AST) was conducted up to 10000 cycles by CVs between +0.6 and +1.2 V vs. RHE. Before and after AST, CV curves were recorded from 0 to +1.2 V vs. RHE at a scan rate of 50 mV s^−1^.

Fuel cell performance was investigated by single cell tests. The membrane electrode assembly was prepared by means of advanced catalyst coated membrane (CCM) technique where the catalyst consisting of active catalysts and Nafion® resin was coated on double sides of the proton exchange membrane^37^,^38^. To prepare the CCM, the Pt/C catalyst was dispersed in a mixture of isopropyl alcohol, deionized water and 5 wt.% Nafion® solution (Du Pont). After ultrasonicated for 20 min, the prepared catalyst ink was sprayed onto Teflon sheets and followed by drying at 100 °C for 1 h. And then, the solidified catalyst layer was transferred onto both sides of the Nafion® NRE◻ 211 membrane (Du Pont) by hot-pressing at 2 MPa and 160 °C for 2 min. The amount of platinum catalyst was controlled to 0.1–0.15 mgPt cm^2^. Gas diffusion layers with porous layers were bought from WUT Energy Co. Single cells, with active area of 5 cm^−1^ × 5 cm^−1^, were assembled by integrating as-prepared CCMs, gas diffusion layers, bipolar plates with straight flow field channels and end plates. Pt loadings at anode/cathode sides correspond to 0.1/0.15 mg cm^−2^. The test was performed with G50 Fuel Cell Test Station (GreenLight) using H_2_/Air (1.5/1.8 stoic) under 150 kPa pressure. The humidity was 50–100% RH at both electrode sides.

## Additional Information

**How to cite this article**: Chen, X. *et al.* Platinized Graphene/ceramics Nano-sandwiched Architectures and Electrodes with Outstanding Performance for PEM Fuel Cells. *Sci. Rep.* **5**, 16246; doi: 10.1038/srep16246 (2015).

## Supplementary Material

Supplementary Information
