# Supplementary material for: Platinized Graphene/ceramics Nano-sandwiched Architectures and Electrodes with Outstanding Performance for PEM Fuel Cells
Source: Sci Rep. 2015 Nov 5;5:16246. doi: 10.1038/srep16246 (PMC4995351; doi:10.1038/srep16246)
Supplement: Supplementary Information [file srep16246-s1.doc]

*Support information*

**Platinized Graphene/ceramics Nano-sandwiched Architectures and Electrodes with Outstanding Performance for PEM Fuel Cells**

Xu Chen, Daping He, Hui Wu, Xiaofeng Zhao, Jian Zhang, Kun Cheng, Peng Wu and Shichun Mu*

State Key Laboratory of Advanced Technology for Materials Synthesis and Processing, Wuhan University of Technology, Wuhan 430070, China

*Corresponding author. E-mail: [msc@whut.edu.cn](mailto:msc@whut.edu.cn)


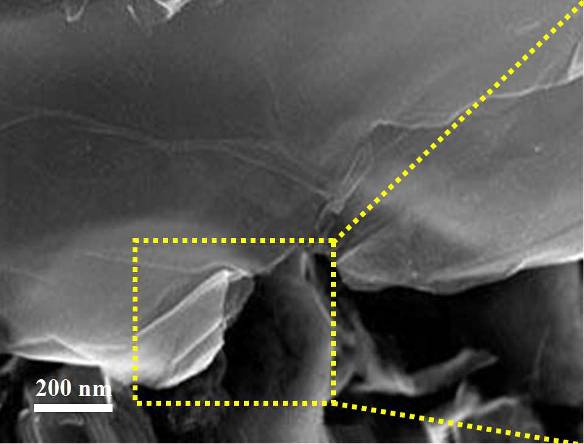


**a**


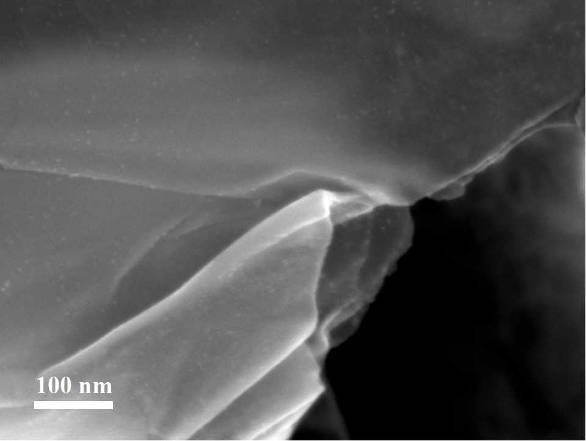


**b**


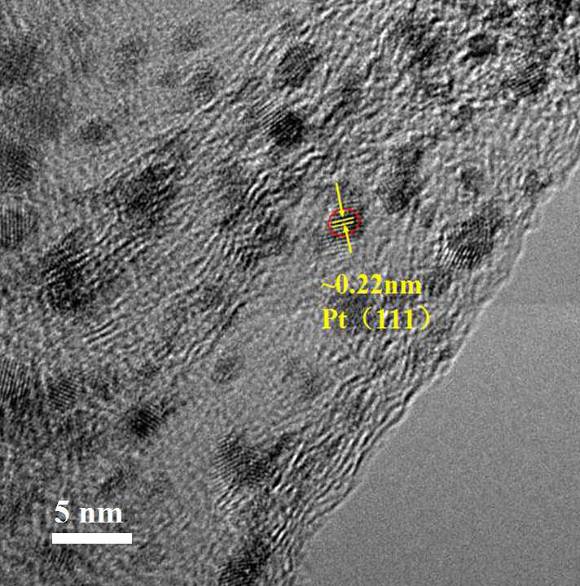


**d**


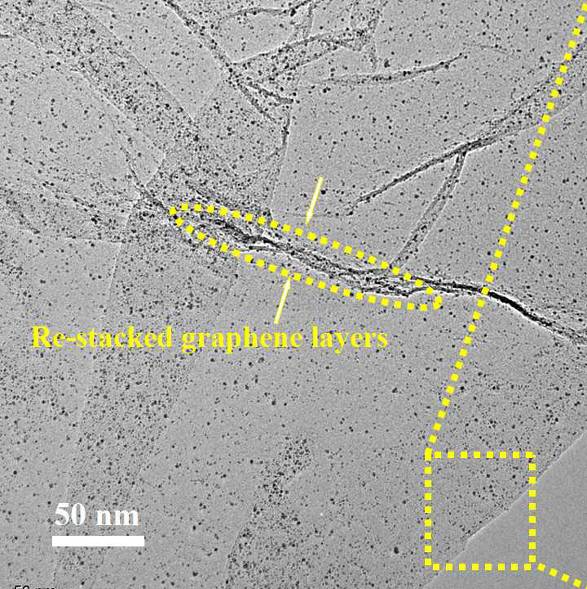


**c**

**Figure S1.** SEM (a,b) and TEM (c, d) images of the Pt/RGO catalyst.


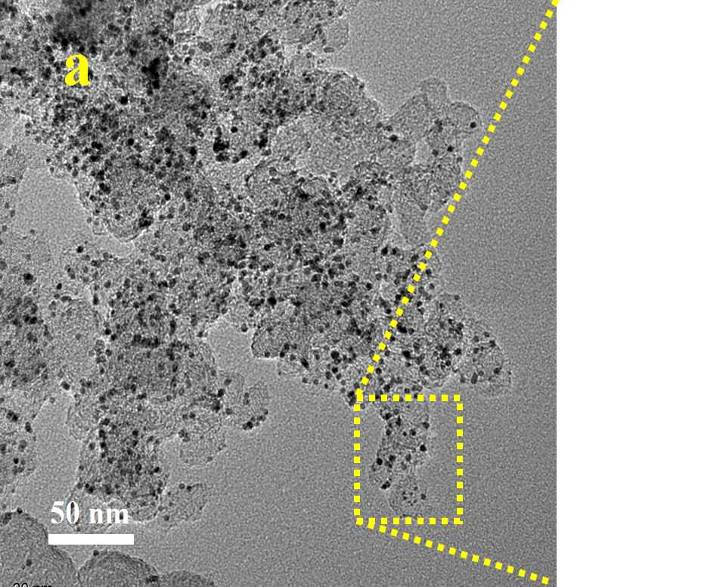

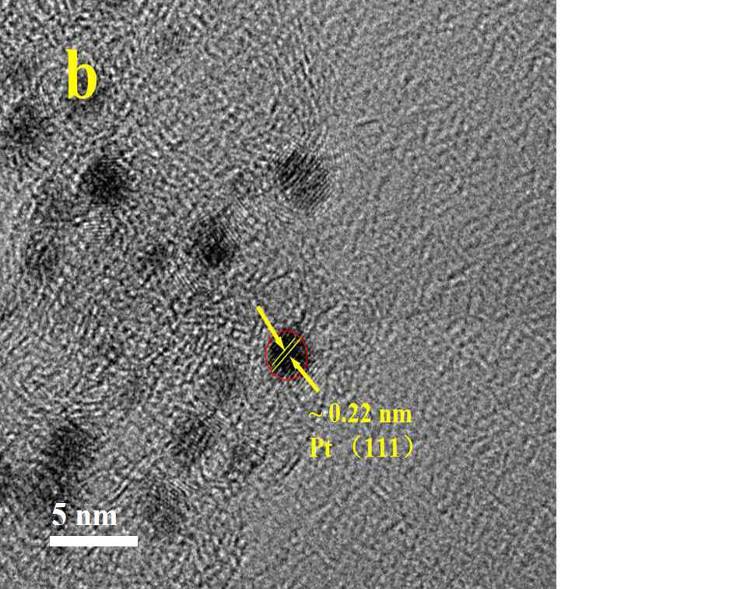


**Figure S2.** TEM images of commercial Pt/C (a and b). The lattice spacing of 0.22 corresponds to Pt (1 1 1) facets. The average particle size of the commercial Pt/C catalyst has a Pt particle size of 2.96 nm.


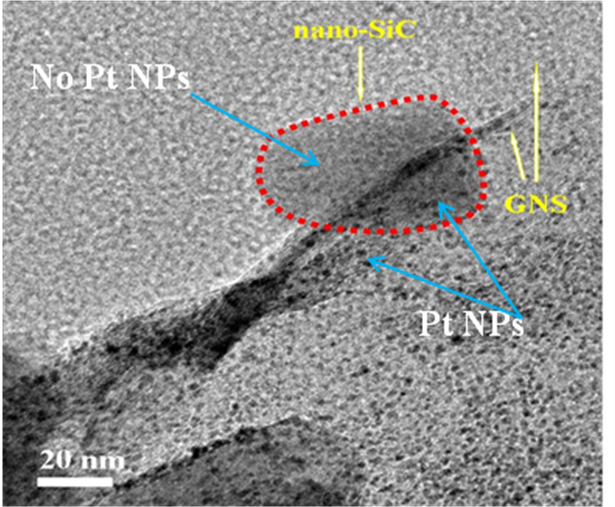


**e**

**Figure S3. A** TEM image partially covered SiC nanoparticle by the Pt dispersed graphene has been observed from

**Figure S4.** Nitrogen adsorption-desorption isotherms of Pt/RGO and Pt-RGO/SiC.


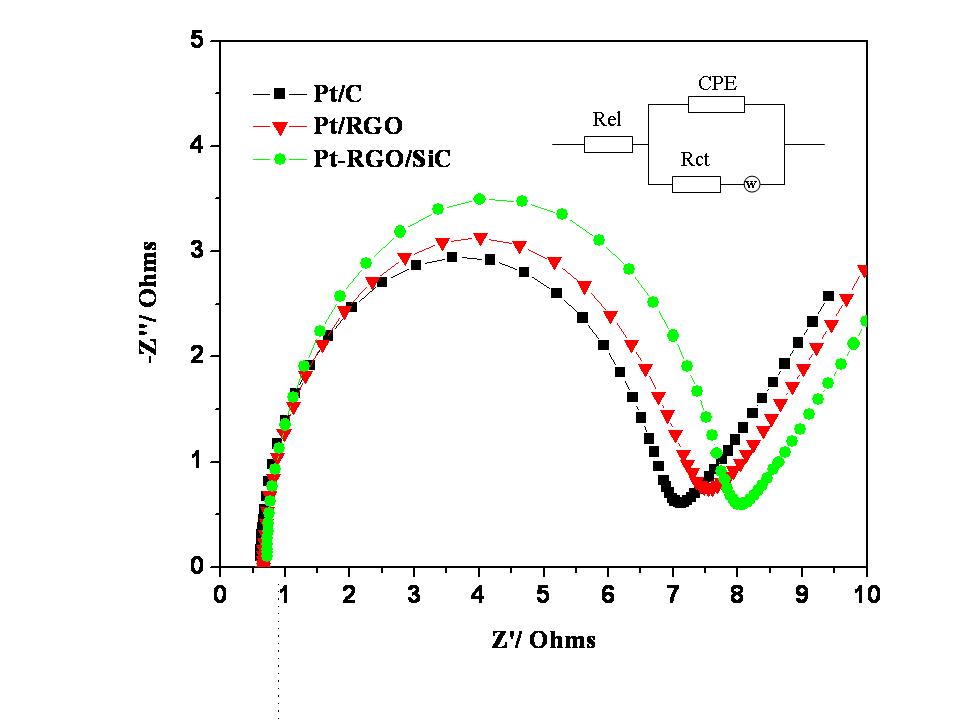


**Figure S5**. Nyquist plots of EIS for Pt-RGO/SiC, Pt/RGO and Pt/C. **A**ll samples contain a partially overlapped semicircle. The charge transfer resistance (Rct) and the uncompensated solution, contact and bulk resistance (R_el_) in different supports can be estimated from the analysis of EIS spectra by using the software ZsimpWin based on an equivalent electrical circuit as shown in the inset of **Figure S4**. Q_dl_ is a constant phase element.


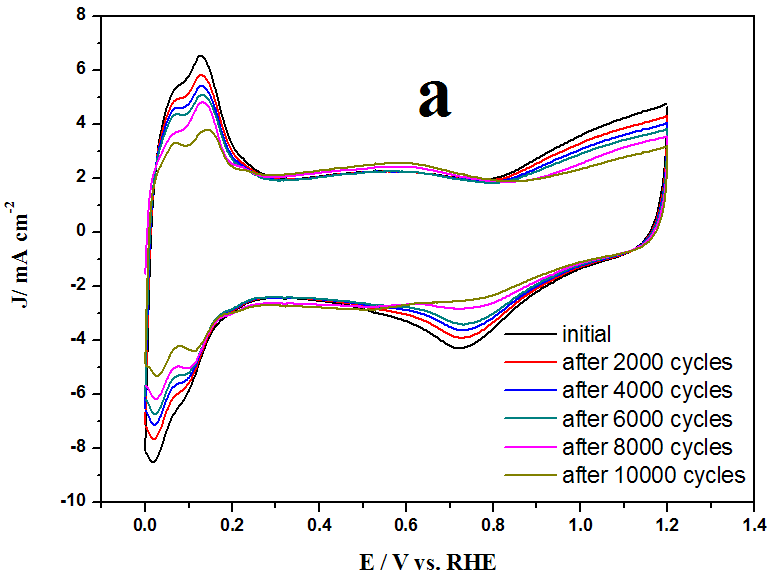

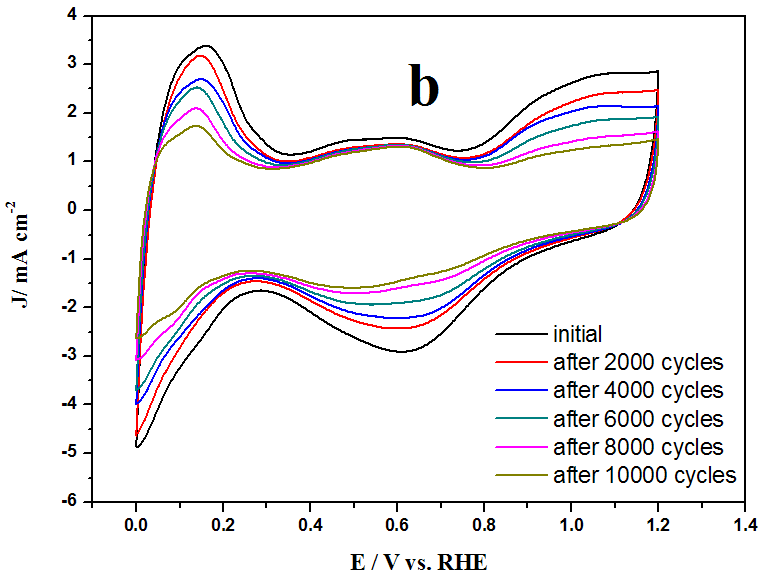

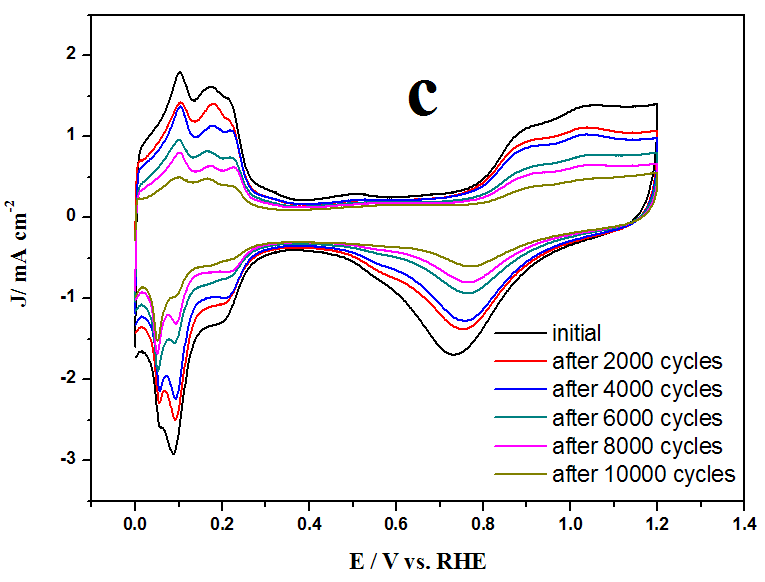


**Figure S6.** CV curves of RGO/Pt/SiC (a), Pt/RGO (b) and Pt/C catalysts (c) by AST in 0.1 M HClO_4_ at room temperatures between +0.6 and +1.2 V vs. RHE at the sweep rate of 100 mV/s as comparisons.

**b**


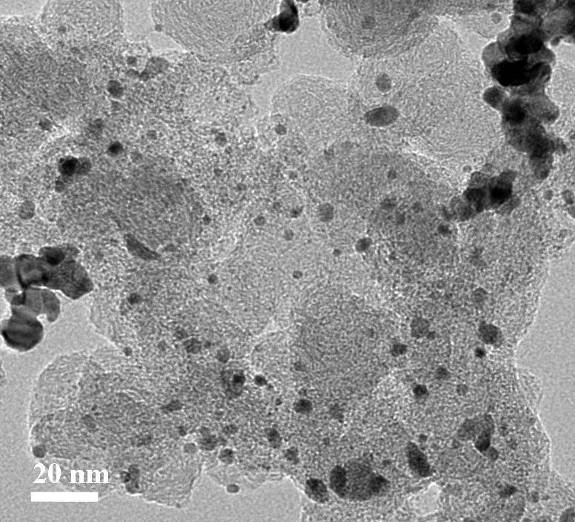


**a**

**Figure S7.** TEM images of the Pt/C (a) catalysts after AST. Pt particle size distributions of Pt/C (b) before (black) and after (red) AST.

To reveal the mechanism of the durability enhancement observed, catalysts were collected by sonicating the GCE in IPA after AST, and their structures were observed by TEM. The particle size distribution of Pt NPs was measured more than 100 particles in each sample. The size of Pt NPs for all the three catalyst samples increases after 4000 CV cycles, indicating the occurrence of agglomeration for metal NPs. As a reference, serious agglomeration of Pt NPS (from 3.0 to 8.0 nm) appears for Pt/C.


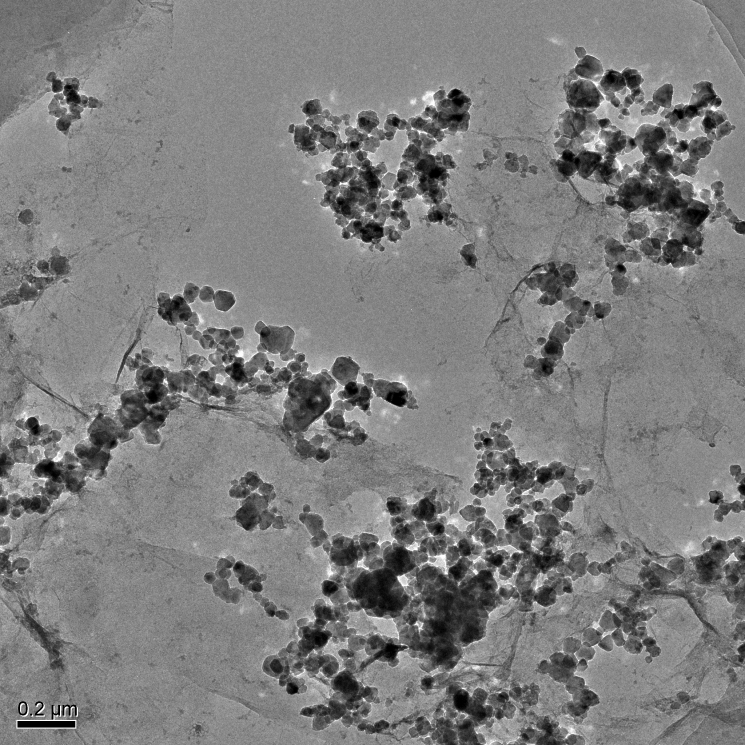


**Figure S8.** A TEM image of the SiC/graphene composite supported Pt NPs by a common synthetic route in solution.
